# The value of post-mortem computed tomography of burned victims in a forensic setting

**DOI:** 10.1007/s00330-018-5731-5

**Published:** 2018-10-01

**Authors:** Henri M. de Bakker, Gijsbrecht H. J. Roelandt, Vidija Soerdjbalie-Maikoe, Rick R. van Rijn, Bernadette S. de Bakker

**Affiliations:** 1 Department of Radiology, Groene Hart Hospital, Gouda, The Netherlands; 2 Department of Radiology, Amsterdam UMC, University of Amsterdam, Amsterdam, The Netherlands; 3 Department of Medical Biology, Section Clinical Anatomy & Embryology, Amsterdam UMC, University of Amsterdam, Meibergdreef 9, 1105 AZ Amsterdam, The Netherlands; 4 Department of Medical Forensic Investigation, Section Forensic Pathology, Netherlands Forensic Institute, The Hague, The Netherlands; 5 Co van Ledden Hulsebosch Center, Amsterdam, The Netherlands

**Keywords:** Burns, Forensic pathology, Radiology, Forensic medicine

## Abstract

**Objectives:**

Fire deaths are challenging fatalities for forensic pathologists, as the main question of whether death was due to the fire or not needs to be answered. In this retrospective study, we assessed whether post-mortem computed tomography (PMCT) has an added value prior to a forensic autopsy of burned victims.

**Methods:**

From 2008 to 2016, a PMCT was performed in 50 burned corpses prior to a complete forensic autopsy. In retrospect, all 50 PMCT scans were systematically assessed by a forensically experienced radiologist, masked from the autopsy reports. Subsequently, the PMCT findings were compared with the autopsy reports.

**Results:**

Heat fractures, contractions and destruction of extremities, subcutaneous emphysema and post-mortem gas collections were easier to detect by PMCT compared to autopsy. Alterations by penetrating and blunt trauma and the presence of foreign bodies were easy to detect by PMCT as well by autopsy. PMCT was, however, not successful in detecting signs of vitality during the fire, detection of superficial thermal injuries and to answer the main question of the forensic autopsy, which is to investigate the cause of death.

**Conclusions:**

PMCT prior to autopsy is a valuable add-on in the post-mortem forensic investigation of burned victims for detection of hidden signs of trauma, gas collections and foreign bodies. However, since PMCT cannot answer the two main questions in forensic examination—determining the cause of death and detecting signs of vitality during the fire—it cannot replace an autopsy.

**Key Points:**

*• Post-mortem CT (PMCT) in burned victims shows hidden signs of trauma.*

*• Foreign bodies and gas collections can easily be detected.*

*• Cause of death and vitality signs cannot be assessed by PMCT.*

## Introduction

Due to the extensive damage that fire can cause to a body, fire deaths are among the most challenging fatalities for pathologists to examine in legal death investigation [1]. Differentiation between ante- and post-mortem injuries, extensiveness of damaged structures, detection of signs of vitality during the fire, as well as the cause of death, are important questions to be answered in these victims. Computed tomography (CT) is known to be of great value for the interpretation of skeletal injuries, as well as the detection of gas collections—two diagnostic criteria that can be difficult to establish during autopsy. The value of post-mortem computed tomography (PMCT) prior to forensic autopsy has been investigated in the past [2–5]. However, no large-scale study is available that compares PMCT findings to autopsy findings in burned victims.

Since 2000, the Radiology Department of the Groene Hart Hospital (GHH) in Gouda, The Netherlands, has collaborated with The Netherlands Forensic Institute (NFI) in The Hague, The Netherlands, in performing PMCT prior to forensic autopsy. The database thereby collected now comprises more than 2,100 forensic radiological cases [6]. Using this database, we retrospectively studied the added value of PMCT prior to autopsy in a case series of 50 burned victims. Three research questions were formulated to compare the findings of PMCT with autopsy:

*Which changes in burned bodies can better be seen on PMCT compared to autopsy?*

*Are signs of vitality during the fire and cause of death detectable by PMCT?*

*Are there ‘typical’ changes in the human body caused by the fire to be seen on PMCT?*

With this in mind, we systematically described the alterations and morphological changes in the bodies affected by fire. Subsequently, we reviewed the autopsy reports of all 50 cases. In this way, we strived to detect the strengths and limitations of PMCT in the forensic investigation of burned bodies. We also describe typical radiological signs that are caused by the effect of prolonged exposure of the body to heat, to increase the awareness for these signs by general radiologists who may encounter such a case.

## Materials and methods

In this retrospective study, we included 50 burned victims collected between 2008 and 2016 in a forensic radiological database, for whom PMCT was performed prior to a forensic autopsy [6]. In the period before 2008, we used mainly conventional X-ray examinations to examine these cases. To be included in this study, the presented body had to have signs of thermal damage and PMCT data had to be available. In some cases, bodies were incomplete due to thermal amputation of the extremities. In one case the cranium was only partly scanned and in one case only a head was presented for investigation. These cases were also included in this study.

Most PMCT scans (*n* = 47) were performed in the GHH, prior to forensic autopsy in the NFI. Two PMCT scans were performed in the University Medical Center Groningen (UMCG) and one in the Maastricht University Medical Center (MUMC+). Since 2009, PMCT scanning in the GHH was performed using a Toshiba Aquilon 64-slice CT scanner (Toshiba Medical Systems Europe, Zoetermeer, The Netherlands). Three older cases were scanned with a Toshiba Aquilon 32-slice CT scanner or a Siemens Somatom 4 (Siemens Healthineers, Erlangen, Germany). The scans were stored and read on a PACS system (Carestream PACS; Carestream Health, Eemnes, The Netherlands).

Before autopsy, the forensic pathologist was informed about the radiological PMCT findings. Forensic autopsy was performed, according to institutional protocol guidelines, by one of the five certified forensic pathologists from the NFI. For all cases an extensive toxicological investigation, including carboxyhaemoglobin levels, and relevant histopathological examination were performed.

An experienced forensic radiologist (H.M.d.B.) and an independent research assistant (G.H.J.R.), both blinded to the original PMCT and autopsy reports, reassessed the 50 PMCT scans. A report template was used to ensure that all cases were reassessed in an equal and complete manner. Working in this way, and including the fact that those 50 cases were reassessed in a period of only a few weeks, enabled the radiologist to identify subtle patterns and new findings related to the exposure of the body to prolonged intense fire. In the assessment of PMCT, we recognised and described typical changes in the human body exposed to fire and we describe them systematically per body region. We compared our findings with the autopsy reports of the forensic pathologists. We examined differences and similarities in the injuries and morphological changes that were found.

## Results

The causes of death of the 50 included victims, as stated by the pathologists, are tabulated in Table 1. In the majority of cases (*n* = 31), death was caused by the fire, through carbon monoxide intoxication (*n* = 21) or by thermal damage to the airways due to inhalation of hot gases (*n* = 10). In a minor group (*n* = 15), death was due to strangulation (*n* = 3), smothering (*n* = 1), blunt trauma (*n* = 4), penetrating trauma (*n* = 6) or disease (*n* = 1). In these cases, the fire was a post-mortem entity. In the remaining four cases (*n* = 4), neither PMCT nor autopsy revealed a cause of death.

**Table 1:** Causes of death based on the autopsy reports

Cause of death	Specifics of cause of death	Certainty of cause of death
Pre-mortem fire effects (n = 31)	- CO intoxication (n = 21) - Thermal damage to airway (n = 10)	Cause of death might have been influenced by blunt force in one case and by drugs and asphyxiation in another case
Fatal neck pressure (strangulation) or airway obstruction (smothering) (n = 4) Fire effects were post-mortem	- Strangulation (n = 3) - Smothering (n = 1)	In all cases cause of death was certain
External blunt force trauma (n = 4) Fire effects were post-mortem	- Beating (n = 3) - Traffic accident (n = 1)	In one case asphyxiation might have contributed to the death due to swelling caused by the beating
Penetrating injury (n = 6) Fire effects were post-mortem	- Stab/cut injuries (n = 3) - Ballistic trauma (n = 3)	In all cases the cause of death was certain
Organ disease (n = 1) Fire effects were post-mortem	- Heart failure (n = 1)	The cause of death was certain
Unknown (n = 4) Fire effects were post-mortem	- Death due to thermal damage was likely, but could not be diagnosed for certain	Forensic autopsy could not determine cause of death

In all cases in which the cause of death was due to carbon monoxide intoxication, thermal damage to the airways or smothering, PMCT appeared not useful in detecting the cause of death. In those cases where the cause of death was due to blunt force or a penetrating trauma, cause of death could be suggested based on PMCT, but autopsy including histology and toxicological investigation was required to provide certainty.

An overview of all major radiological findings is illustrated in Table 2. Multiple affected body regions can be present in one case.

**Table 2:** Findings using PMCT in burned victims

Cases ^a^	Specifics of damage	Causes of damage	Other findings
Cranial/facial burns (n = 24)	Thermal soft tissue defects without skeletal damage: (n = 4)	All caused by fire	Damaged cerebrum and cerebellum (n = 17) Dural rupture (DR) with herniation (H) of brain tissue (Fig. 1a and b) (n = 16) *Heat haematoma* (Fig. 1b and c) (n = 11) *Heat haematoma* with DR + H (Fig. 1b) (n = 5) Foreign bodies (n = 8) Heat fracture, *split diploë* (Fig. 1d-f) (n = 5) Intracranial air (n = 4)
	Thermal soft tissue defects with skeletal damage: (n = 9)	Bone damage caused by: Fire (n = 5) Blunt force ^b^ (n = 4)	
	Thermal soft tissue defects with loss of skeletal structures: (n = 11)	Impossible to determine ^c^	
	Skeletal damage without thermal soft tissue defects: (n = 3)	Bone damage caused by: Blunt force (n = 2) Penetration (n = 1)	
Neck burns (n = 23)	Thermal soft tissue defects without skeletal damage: (n = 10)	All caused by fire	Fractured spinal column (n = 3) Bony fragments dislocated by projectile (Comet tail) (n = 1) Fractured hyoid bone
	Thermal soft tissue defects with skeletal damage: (n = 5)	Bone damage caused by: Fire (n = 4) Blunt force (n = 1)	
	Thermal soft tissue defects with loss of skeletal structures: (n = 8)	Impossible to determine	
	Skeletal damage without thermal soft tissue defects: (n = 7)	Bone damage caused by: Blunt force (n = 6) Penetration (n = 1)	
	Soft tissue defects not caused by fire: (n = 5)	Swelling (n = 2) Penetration (n = 3)	
Thorax burns (n = 32)	Thermal soft tissue defects without skeletal damage: (n = 8)	All caused by fire	Fluid in chest cavity (n = 19, of which 16 cases concerned blood) Pneumothorax (n = 9) Pneumomediastinum (n = 9) Foreign bodies (n = 5) Fractured spinal column (n = 4) Subcutaneous emphysema (n = 3) Fractured scapula (n = 3) Damaged heart (n = 2) Pneumopericard (n = 2) Air inside heart chambers (n = 2) Fluid in the pericard (n = 2) Burned through spinal column (n = 1)
	Thermal soft tissue defects with rib fracture: (n = 5)	Bone damage caused by: Old fracture ^d^ (n = 2) Blunt force (n = 1) Ballistic trauma (n = 2)	
	Thermal soft tissue defects with loss of rib structures: (n = 19)	Impossible to determine	
	Rib damage without thermal soft tissue defects: (n = 5)	Bone damage caused by: Penetration (n = 2) Old fractures (n = 2) Blunt force (n = 1)	
Abdomen burns (n = 34)	Thermal damage without an opening to the abdominal cavity: (n = 15)	All caused by fire	Air inside organs (n = 19) Thermally damaged hip bones (n = 16) Foreign bodies (n = 5) Burned sacrum (n = 2) Bullet trajectory (n = 1) Fractured spinal column (n = 1) Abdomen burned completely through (n = 1)
	Thermal soft tissue defects with an opening to the abdominal cavity: (n = 19)	Burned organs: Bowels (n = 8) Liver (n = 5) (4 with dense border, 1 liver missing)	
	Damage to internal organs not related to burns: (n = 6)	Liver (n = 1) ballistic trauma Kidney (n = 3) (2 missing, 1 bleeding) Spleen (n = 2) (both missing)	
Upper extremity burns (n = 40)	Thermal soft tissue defects without skeletal damage: (n = 17)	All caused by fire	Pugilistic attitude (n = 21) Thermal amputation (n = 20) Not completely imaged (n = 5) Arms fixated pre-mortem (n = 2) Dislocated elbow (n = 1) Dislocated wrist (n = 1) Foreign bodies (n = 1)
	Thermal soft tissue defects with skeletal damage: (n = 3)	All caused by fire	
	Thermal soft tissue defects with loss of skeletal structures: (n = 20)	Impossible to determine	
Lower extremity burns (n=36)	Thermal soft tissue defects without skeletal damage: (n = 19)	All caused by fire	Thermal amputation (n = 15) *Luxation* of ankle (Fig. 5a) (n = 4) Proximal displacement of patella (Fig. 5) (n = 6) Foreign bodies (n = 2) *Luxation* of knee (n = 1) Not completely imaged (n = 1) Ankle arthrodesis Prosthetic hip
	Thermal soft tissue defects with skeletal damage: (n = 2)	All caused by fire	
	Thermal soft tissue defects with loss of skeletal structures: (n = 15)	Impossible to determine	
	Skeletal damage without thermal soft tissue defects: (n = 1)	Blunt force	

^a^Multiple affected body regions can be present in one case

^b^Blunt force was seen as the cause of damage when no soft tissue defects matching thermal or penetrating injury was found

^c^Due to the extent of fire damage caused to the area it is impossible to determine if other forces were applied

^d^A fracture was designated as old when a callus was present

### PMCT findings

#### Head

**Fig. 1:**
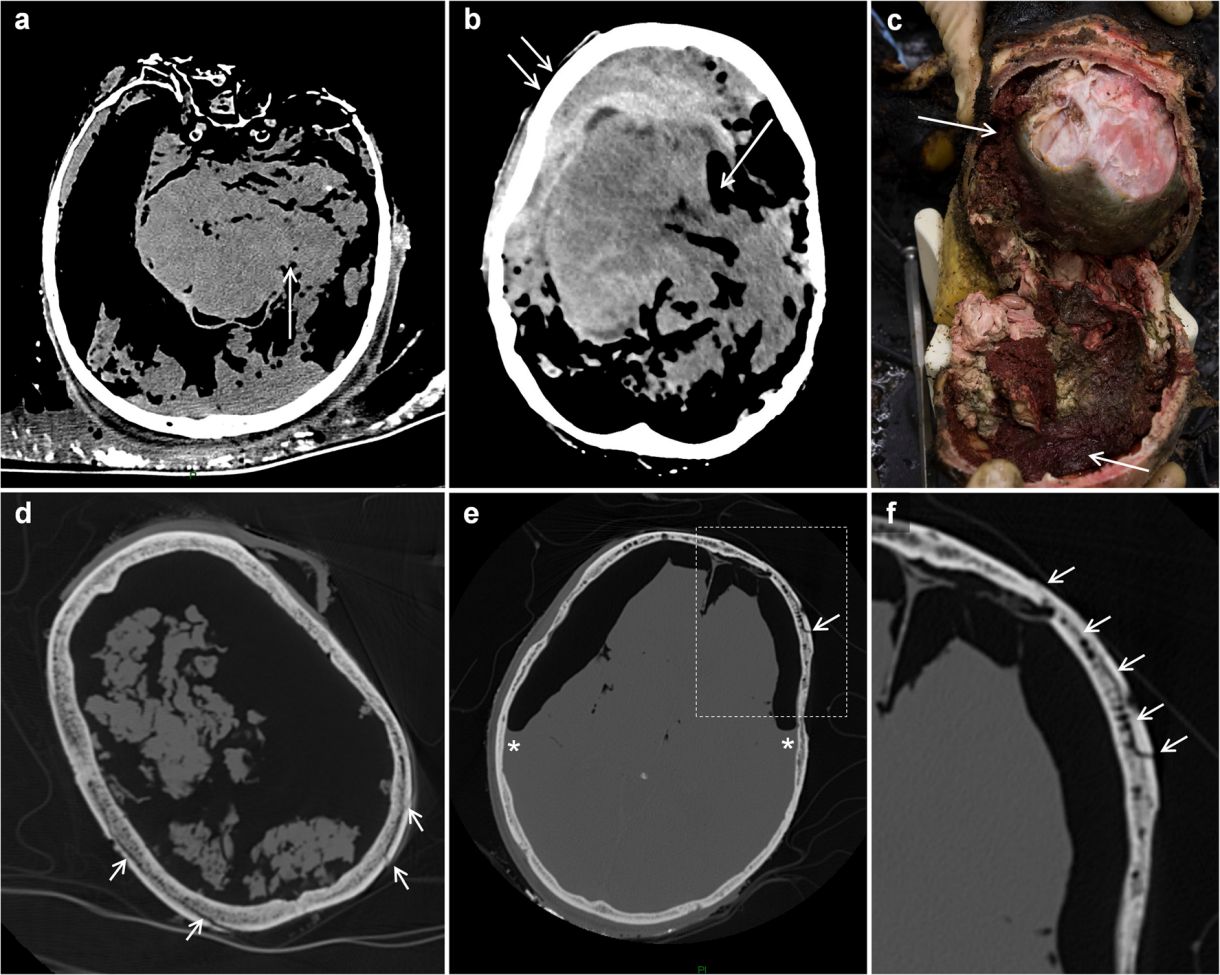
Typical post-mortem CT findings in the head exposed to fire. **a**, **b** Axial images. In both cases, a dural tear and herniated brain tissue can be seen (*single arrow*). The case in (**b**) shows also an intracranial *heat haematoma* (*double arrow*). **c** Picture of an opened skull with *heat haematoma* (*arrows*) during autopsy. **d**, **e** Axial images. In both cases, heat fractures can be seen in the outer table (*arrows*) on locations where all soft tissue are burned away, resulting in separation of inner and outer table (*split diploë sign*). The case in (**e**) shows also a bilateral intracranial *heat haematoma*; *air-fluid level. **f** Enlarged section of (**e**). The *arrows* indicate the *split diploë sign*

Burning effects on the head were seen in 24 cases. In 17 cases, the skull and intracranial structures were damaged. Herniation of brain tissue through the dura (dural tear) was observed in 16 cases, 5 times in combination with a ‘*heat haematoma*’ (Fig. 1a and b). In total, 11 cases with *heat* haematomas were observed (Fig. 1b and c). In five cases, heat fractures of the skull were observed where the soft tissues covering the skull were completely burned away. We called this typical splitting of the inner and outer table of the skull the ‘*split diploë sign*’ (Fig. 1d–f). In cases where both the inner and outer table were fractured and part of the skull was missing, *heat haematoma* was absent. In two cases of blunt force trauma, facial soft tissue swelling (subcutaneous oedema) was present. In one case, the cranium was only partly scanned.

#### Neck

**Fig. 2:**
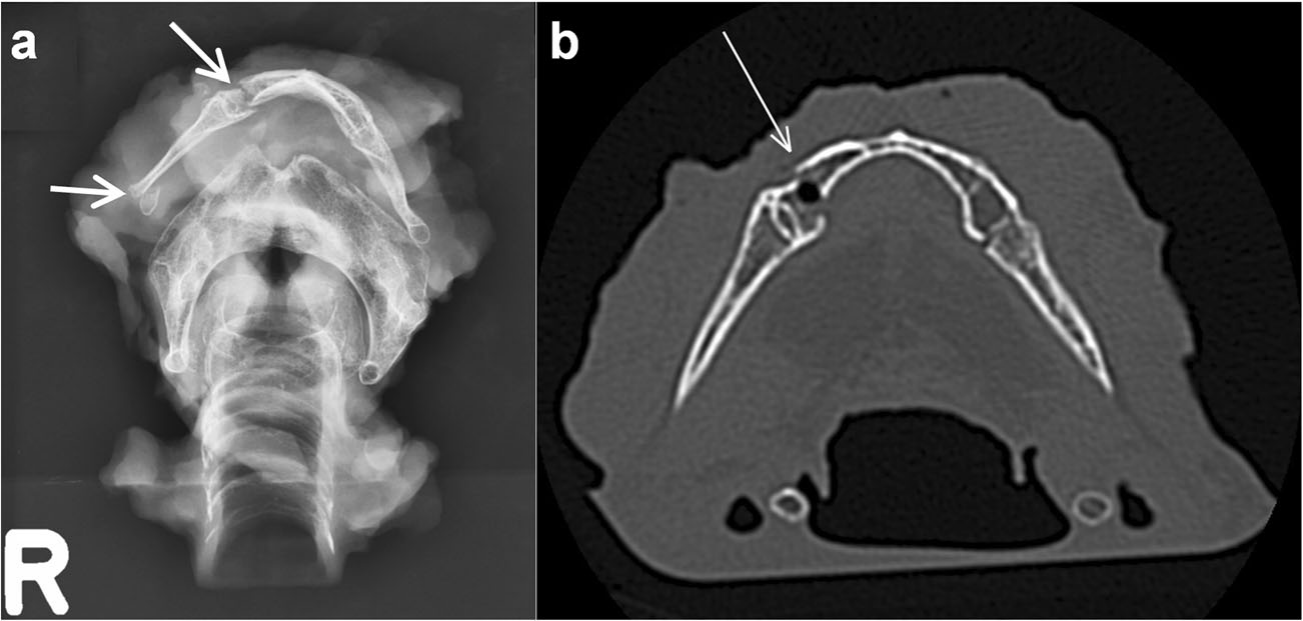
Radiology of the explanted hyoid-larynx complex from a body exposed to fire.** a** X-ray shows fractures of the hyoid bone body and the greater horn on the right side (*arrows*). **b** Same case as in (**a**). Axial CT image of the body and greater horns of the hyoid bone. The fracture of the hyoid body is indicated by the *white arrow*. Histology showed haematomas at the fracture sites, which indicate that the person was alive when trauma to the neck was sustained

In 23 cases, there was damage to the neck. On PMCT we found three cases with a fractured spinal column. One case of ballistic trauma showed a classic comet tail, i.e. fragments of the cervical spine dislodged by the projectile. In one case, a hyoid bone fracture was noted on the PMCT. The excised hyoid-larynx complex showed fractures of the hyoid bone body and greater horn on specimen imaging (X-ray and CT) (Fig. 2). Histology revealed haematomas at both fracture sites, suggesting an ante-mortem trauma mechanism.

#### Thorax

**Fig. 3:**
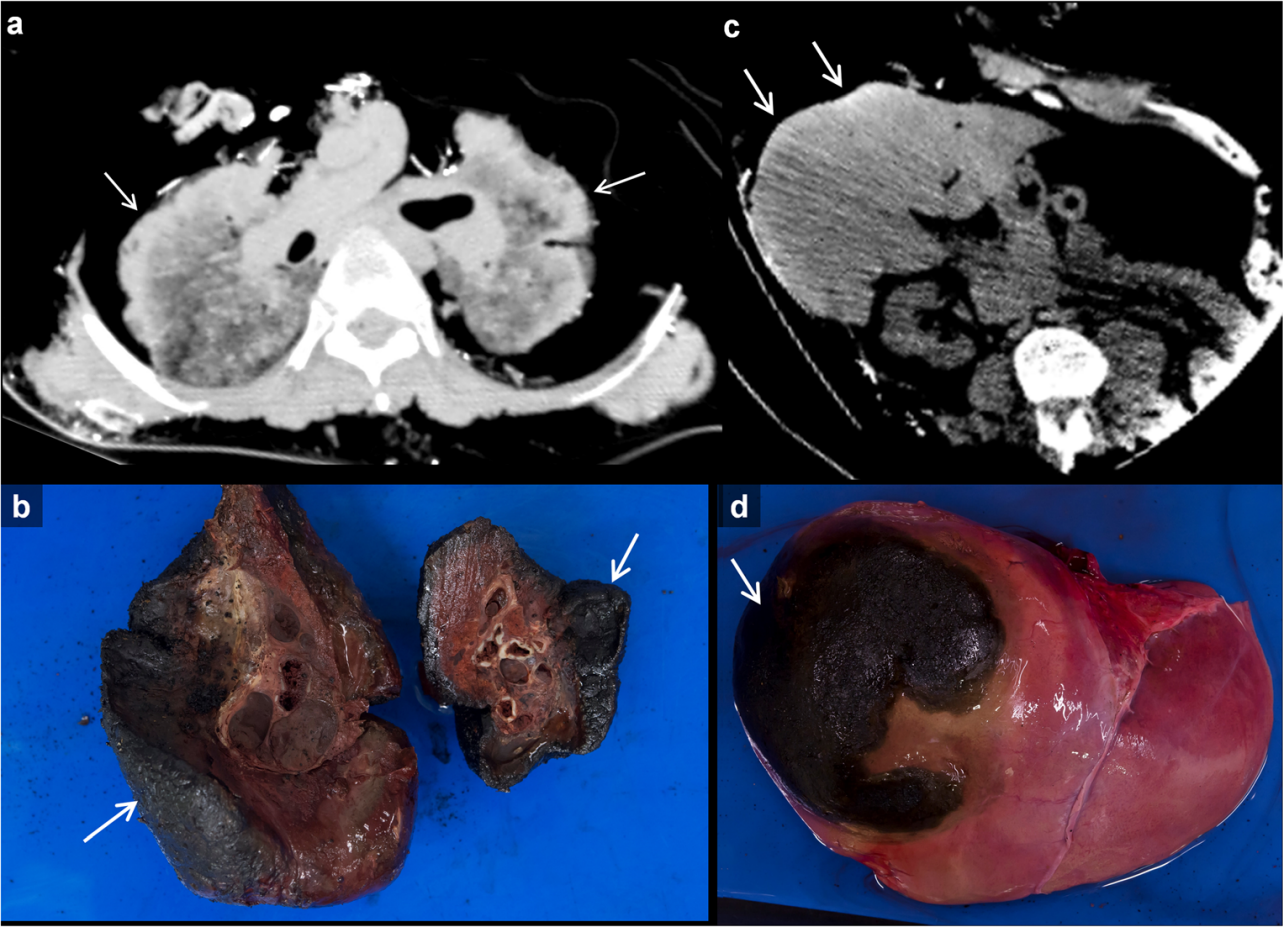
Examples of *dense border sign* on CT and at autopsy.** a** Axial image of a burned thorax. A ‘*dense border sign’* can be seen at the surface of the lung that was exposed to fire (*arrows*). This is due to shrinkage and loss of fluid of the exposed tissue [3]. Note how the lungs are relatively intact in contrast to the absent burned surrounding structures. **b** Pathological specimen of the lung from the case presented in (**a**), showing a partly burned surface (*arrows*) that was in direct contact with the fire. **c** Axial image of a burned abdomen. A subtle *dense border sign* can be seen at part of the liver surface that was exposed to fire (*arrows*). *See* (**d**) for the pathological specimen of this case. **d** Pathological specimen of the liver from the case presented in (**c**), showing a partly burned surface (*arrow*) that was in direct contact with the fire, and a relatively normal part of the liver that was covered and protected from the fire by the body wall

Thirty-two cases showed damage to the thorax. Two cases had old rib fractures with callus formation. Furthermore, two peri-mortem rib fractures caused by blunt force and two rib fractures due to ballistic trauma were found. Pleural effusion was present in 19 cases. In 16/19 cases, the effusion was determined to be haemorrhagic based on high CT attenuation and dependent sediment. In 17 cases, an opening through the chest wall was the result of fire. High attenuation of the lung surface (so-called '*dense border sign*') was present in cases with direct exposure of the lung surface to fire (Fig. 3a and b). In nine cases (seven unilateral, two bilateral), a pneumothorax was found with an intact thoracic wall. In one of these cases with pneumothorax, rib fractures were present; another was found in a case with ballistic trauma. Damage to the heart was found in a case with thermal damage of the thorax and in one case with ballistic trauma. Four cases contained a foreign body in the thoracic area, i.e. a nasogastric tube, cardiac implantable electronical device wires without a device present (the chest wall area was heavily burned so it could have fallen out of the body), three bullets in one case and one bullet in another case.

#### Abdomen

**Fig. 4:**
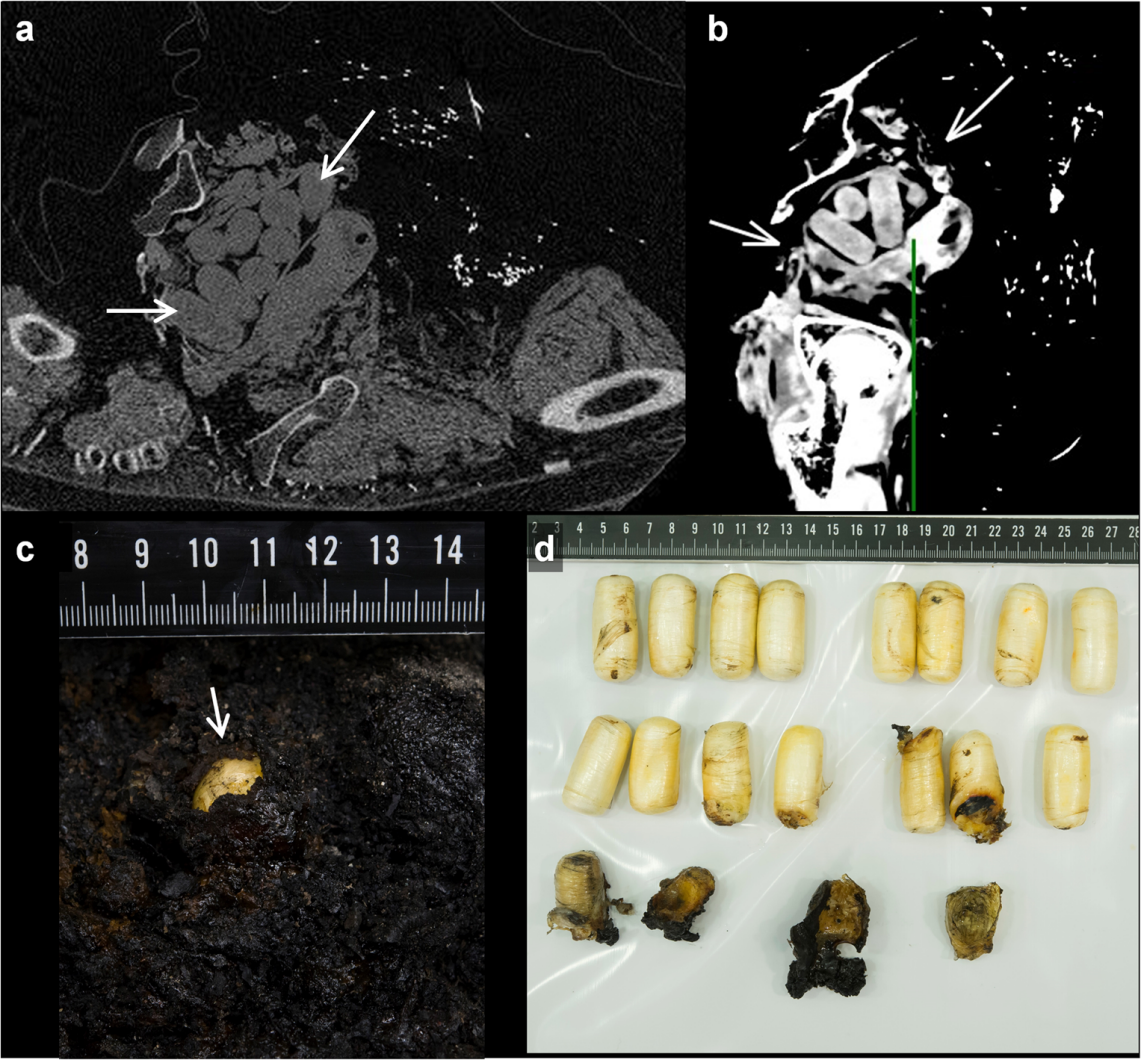
Drug packages in a burned body. **a** Axial image of a body almost completely destructed by fire. Note the relative sparing of the drug packages (*arrows*). **b** Reconstructed sagittal image of the same case as in (**a**). Drug packages are indicated by *arrows*. **c** Image taken during autopsy of the carbonised body of the victim presented in (**a**) and (**b**). Note the light-coloured foreign body as small detail in the blackened tissue at autopsy. **d** Overview of the relatively unharmed drug packages that were recovered from the carbonised body presented in (**a**–**c**) during autopsy

Damage to the abdomen was seen in 34 cases. The abdominal wall was completely destroyed by fire in 19 cases. The abdominal organs, however, were often relatively well preserved in these bodies. Twelve cases showed abdominal organ damage, out of which three had exclusive damage to the bowel. One case showed a ‘*dense border sign*’ of part of the liver exposed to fire (Fig. 3c and d). In five cases, foreign bodies were identified in the abdomen—i.e. a common bile duct stent, four bullets (of which one was embedded in the pubic bone), drug packages in the bowels (Fig. 4) and an implantable cardiac defibrillator, which likely slid into the abdominal cavity due to significant destruction of the thorax. The latter is another case as described in the thorax section.

#### Extremities

The majority of cases (40/50) demonstrated burns of the upper extremity. In nearly half of the cases (*n* = 17) only soft tissues were damaged. Three had burns with a fracture of a bone (one of the ulna and two with a fracture of the proximal phalanx of the fifth digit). Twenty cases of thermal amputation in varying degrees were noted—i.e. seven cases where at least one finger was lost, seven amputations of (or part of) the lower arm and six at the level of the upper arm. Furthermore, in 21 cases a typical pugilistic posture was found. In one case both the wrist and elbow were luxated. In two cases the arms were fixated behind the back, where wires were present around the wrists. In five cases parts of the arms were not scanned due to the flexion deformities in relation to the limited bore diameter of the CT scanner.

**Fig. 5:**
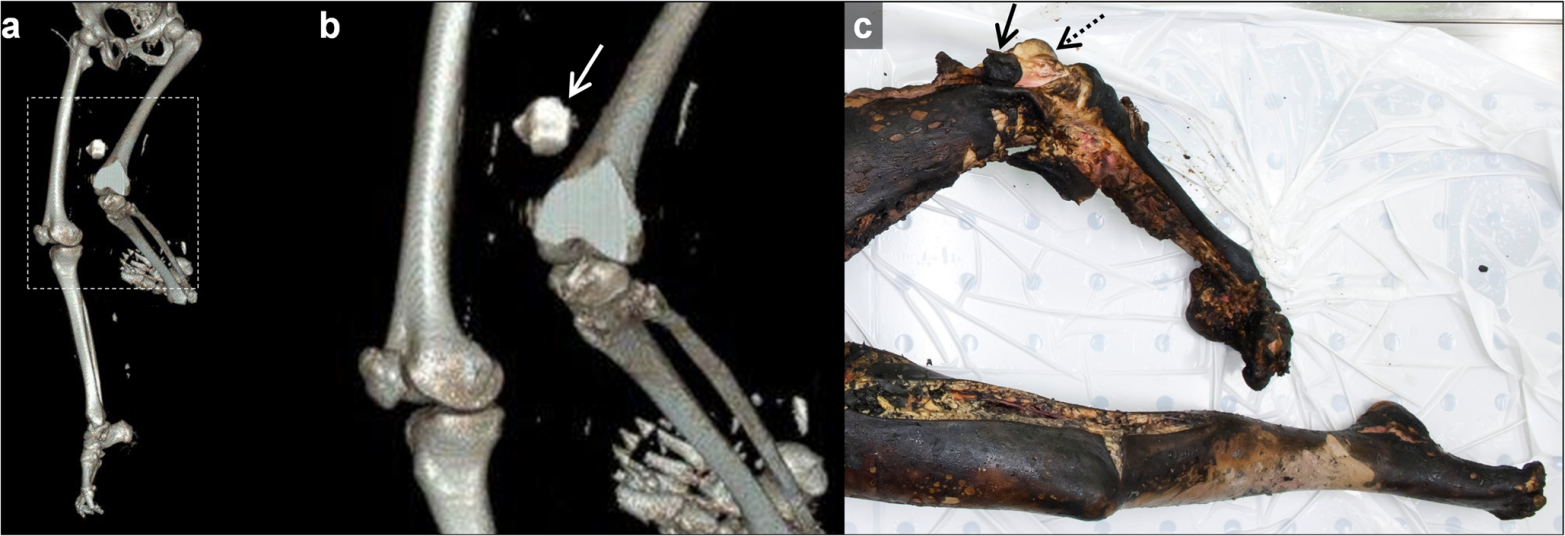
Typical proximal displacement of the patella in the left knee exposed to fire.** a** Anteroposterior 3D reconstruction of a CT of the lower extremities of a body exposed to fire, presenting patellar displacement. **b** Enlarged part of the 3D-CT scan in (**a**). Note the proximal displacement of the patella of the left knee (*arrow*). The patellar ligament is burned through or ruptured by the force of the shrinking rectus femoris muscle. **c** The proximally displaced and blackened left patella (*black arrow*) could be identified at autopsy. Its normal location is indicated by the *dashed arrow*. Typical flexing of the joints in legs and feet that were exposed to fire can be appreciated. Note also the coverage of the right patella by soft tissues, which prevents the patella from proximal displacement

Thirty-six subjects had burns of the lower extremity. Fifteen instances of various degrees of thermal amputation were found—i.e. two amputations of the foot, five of the lower leg and eight of the upper leg. *Luxation* of the ankle in four cases (Fig. 5a), of the knee in one case and a proximal displacement of the patella in six cases were also observed (Fig. 5). Additionally, an arthrodesis of the ankle was seen in one case and a prosthetic hip in another. In one case the leg was not completely imaged due to the flexion deformities.

### PMCT versus autopsy

**Fig. 6:**
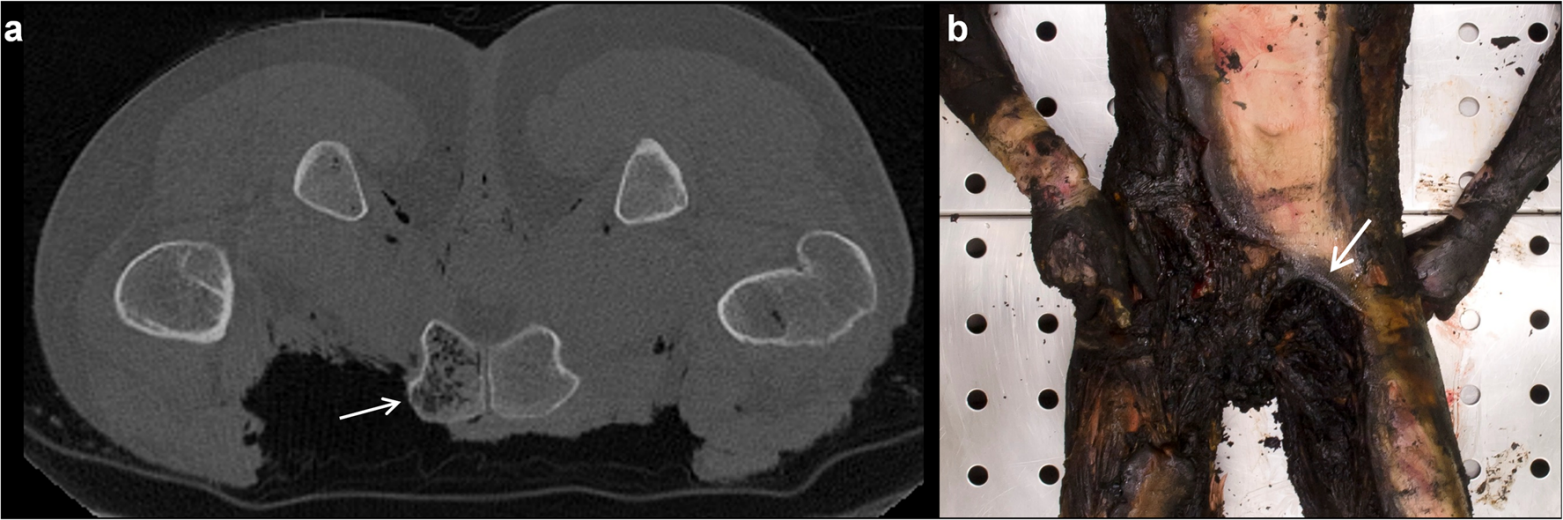
Example of *mottled lucencies* in a burned body.** a** Axial image of the pubic bone, scanned in prone position as it was found at the crime scene. The left side of the pubic bone, not covered by soft tissues, contains *mottled lucencies* inside of the bone (*arrow*), whereas the right pubic bone appears normal [3]. **b** The same case as in (**a**) at autopsy. Ventral view of the pelvic region. Forearms and hands are also visible. The partially intact abdominal skin of the victim suggests a prone position during the fire. Note the heavily burned left groin (*arrow*) compared to the right groin that is still more or less covered by soft tissues

When comparing PMCT to autopsy findings, some discrepancies were noted. PMCT scans revealed subcutaneous emphysema in three cases and damage of skeletal structures in nine cases (three of facial skeletal structures, two of the ribs, three of the scapula and one of a spinous process), which were absent from the autopsy reports. The PMCT finding of *mottled lucencies* found in bones with direct exposure to fire was not listed in the autopsy reports (Fig. 6). In contrast, autopsy revealed soot and thermal damage in the airways in 19 cases, damage of the internal organs in six (two caused by thermal damage and three caused by penetrating trauma), one ruptured aorta and nine cases of soft tissue defects such as cuts and lacerations, charring, skin splitting and other superficial changes to the skin that were not seen on PMCT. In one case, histological examination revealed that a fatal heart disease was the cause of death.

## Discussion

PMCT scans showed extensive morphological changes to the bodies of burned victims. Using PMCT, the radiologist was able to consistently detect damage to and loss of soft tissue, damage to bones, and the location of gas collections and foreign bodies (Fig. 4).

We found some recurring typical patterns on PMCT scans. The first were radiolucent areas inside the bones described as *mottled lucencies* (Fig. 6a). These small intraosseous gas collections in the marrow spaces were found in bones that lost their soft tissue cover and were directly exposed to the heat of the fire and subsequent dehydration, as described before by Levy et al [3]. It can be distinguished from general decomposition by the lack of these changes in the other bones, still covered by soft tissue.

In addition, we found multiple cases of intracranial air when the soft tissues of the head were lost through the fire, but the skull itself remained intact and there were no other signs of putrefaction. Furthermore, splitting of the inner and outer table of the skull with lifting of the outer table (*split diploë sign*) was seen in some skulls exposed to fire (Fig. 1d–f). Thermal violence to the head caused herniation of brain tissue through a tear in the shrunken dura (Fig. 1a and b). These patterns of heat fracturing and herniation of brain tissue were also described before [7]. The *heat haematoma* is defined by a unilateral or bilateral pool of blood in the extradural space of a skull subjected to heat [7]. It can be distinguished from an ante-mortem extradural haematoma by collecting specimens at autopsy, followed by toxicological investigation [7]. There should be little or no carboxyhaemoglobin in the extradural haematoma if the victim suffered head injury before the fire started.

Furthermore, shrinkage of internal organs due to heat is a well-documented effect of thermal damage [3]. We found a typical *dense border sign* (Fig. 3a and c) in cases where lung, liver or brain was directly exposed to fire. The heat causes shrinkage and dehydration of the exposed tissue [3]. In these cases the forensic pathologist found thermal damage to the surface of the tissues and organs in the sense of blackening and stiffness of the surface (Fig. 3b and d). To our knowledge, this sign has not been reported before.

Muscles and organs did not burn easily, but retracted or shrunk instead. Tissues such as skin, tendons, bones and fat, however, appeared much more vulnerable for thermal damage. Bone structures with a limited soft tissue shielding, such as the skull, wrists, knees, fingers, toes and hips, were more likely to burn through than extensively covered bones, such as the femur. Another reason for the varying degrees of thermal destruction might be the position the body assumes when exposed to heat [8]. The arms take on a pugilistic attitude and the legs flex (Fig. 5) [3, 9]. This position causes certain areas, such as the wrists and knees, to be pointed towards the fire and to receive more oxygen, due to their distance from the body and ground, which facilitates tissue destruction by the fire [8]. Dislocation of joints is likely caused by loss of support from soft tissues combined with the force of the retraction of these tissues caused by heat [3, 9]. The force of retracting muscles could also be the cause of the proximal dislocation of the patella (*n* = 6) (Fig. 5). The patellar ligament burned through or ruptured by the force of the shrinking and retracting rectus femoris muscle.

Some autopsy findings were not detectable using PMCT. Findings that were consistently not found using PMCT were superficial thermal injuries, such as blistering and charring of the skin and organs, and the presence or absence of soot and thermal damage in the airways. These airway findings, in combination with a measurement of blood carboxyhaemoglobin, are essential to prove vitality during the fire and to determine it as a cause of death [1, 2]. Autopsy also showed bruising around the larynx, which might indicate strangulation. By histological investigation a pathological substrate of a heart disease was found in one case. This means that forensic autopsy followed by toxicological and histological investigation remains essential to determine the cause of death.

One benefit of PMCT is that data which we used in this retrospective study were similar as the data we acquired prior to autopsy. When questions arise later during the forensic/legal investigation, similar reviews can be added to forensic autopsy findings. The addition of cross-sectional imaging to forensic autopsy allows the radiologist and pathologist to view post-mortem anatomy in two and three dimensions without dissection and to demonstrate those images to laymen in court in a non-confronting manner instead of using, sometimes disturbing, autopsy images.

Another advantage of PMCT is the ability to explore internal body parts which are not routinely examined by the forensic pathologist in The Netherlands during autopsy (e.g. the face and the extremities), as those areas are not incised routinely. A number of fractures found using PMCT were missed during autopsy. These fractures were often subtle and hard to uncover, like in heavily thermally damaged body regions. Also, subtle radiopaque details like metal splinters can easily be discovered. The informed pathologist can deviate from routine autopsy to examine essential details that without the use of PMCT prior to autopsy would have been missed. Lastly, since implants are perfectly visible on PMCT, this is of great use in victim identification [4].

The main limitation of this retrospective study was the fact that the pathologists were not masked from PMCT findings during autopsy. This adversely affects some benefits of PMCT because more lesions or clues were found during the autopsy following the PMCT findings. A refinement of the present study could be that in a prospective study the forensic pathologist will be informed about the PMCT findings at the near end of the autopsy. Only then the added value of the PMCT preceding autopsy could be fully exploited. Another limitation was that in some cases parts of the extremities could not be scanned due to flexion deformities in relation to the limited bore diameter of the scanner. With the newer wide bore CT scanners, this problem will be mostly overcome.

We conclude that PMCT has its value in forensic investigation of burned bodies in the assessment of soft tissues, skeletal structures and localisation of foreign bodies and gas collections. Furthermore, PMCT showed typical changes by body parts exposed to fire, e.g. *split diploë sign* of the tables of the skull, intracranial *heat haematoma*, *dense border sign* of brain, liver and lung, *mottled lucencies* in bones, and joint *luxation*. PMCT excels in finding injuries in areas not normally dissected during forensic autopsy. However, the main question in post-mortem investigation of burned victims as determination of signs of vitality during the fire and cause of death could only be answered by autopsy including histological and toxicological investigation. We conclude that PMCT prior to autopsy is a valuable add-on in the post-mortem forensic investigation of burned victims.

## Abbreviations

PMCT: Post-mortem computed tomography
